# 

**Published:** 1861-09

**Authors:** 


**N. S. DAVIS, M. D., Editor.---FRANK W. REILLY, M. D., Ass't Editor.**

A MONTHLY JOURNAL,

DEVOTED TO THE

**EDUCATIONAL, SCIENTIFIC, AND PRACTICAL INTERESTS**

OF THE

MEDICAL PROFESSION.

The EXAMINER will be issued during the first week of each month, commenceing with January, 1860. Each number will contain 64 pages of reading matter, the greater part of which will be filled with such contents as will directly aid the practitioner in the daily practical duties of his profession.

To secure this object fully, we shall give, in each number, in addition to ordinary original articles, and selections on practical subjects, a faithful report of many of the more interesting cases presented at the Hospitals and College Cliniques. While aiming, however, to make the EXAMINER eminently practical, we shall not neglect either the scientific, social, or educational interests of the profession. It will not be the special organ of any one institution, society, or clique; but its columns will be open for well-written articles from any respectable member of the profession, on all topics legitimately within the domain of medical literature, science and education.

Terms, $2.00 per annum, invariably in advance.

